# Associations between apparent diffusion coefficient and electromyography parameters in myositis—A preliminary study

**DOI:** 10.1002/brb3.958

**Published:** 2018-03-30

**Authors:** Hans‐Jonas Meyer, Alexander Emmer, Malte Kornhuber, Alexey Surov

**Affiliations:** ^1^ Department of Diagnostic and Interventional Radiology University of Leipzig Leipzig Germany; ^2^ Department of Neurology Martin‐Luther University Halle‐Wittenberg Halle Germany

**Keywords:** apparent diffusion coefficient, diffusion‐weighted imaging, electromyography, myopathy, myositis

## Abstract

**Objective:**

MRI is widely used in several muscle disorders. Diffusion‐weighted imaging (DWI) is an emergent imaging modality sensitive to microstructural alterations in tissue. The apparent diffusion coefficient (ADC) is used to quantify the random motion of water molecules. Electromyography (EMG) is a clinically used diagnostic tool in myositis. The aim of this study was to elucidate possible associations between ADC values and EMG findings in myositis patients.

**Method:**

Seven patients (eight investigated muscles) with myositis (mean age 51.43 ± 19 years) were included in this study. The diagnosis was confirmed by histopathology in every case. DWI was obtained with a 1.5‐T scanner using two b‐values 0 and 1000 s/mm². In all patients, a needle electromyography (EMG) was performed within 3 days to the MRI. The following EMG parameters were studied: motor unit action potential (MUAP) amplitudes and durations, as well as pathological spontaneous activity. Spearman's correlation coefficient was used to analyze associations between investigated parameters.

**Results:**

The estimated mean ADC_mean_ value was 1.51 ± 0.29 × 10^−3 ^mm²/s, mean ADC_min_ was 1.28 ± 0.27 × 10^−3 ^mm²/s, and mean ADC_max_ was 1.73 ± 0.28 × 10^−3 ^mm²/s. Correlation analysis identified significant associations between ADC_mean_ and duration of the MUAP (p* *= .78 P = .0279) and between ADC_min_ and duration of the MUAP (p = .85, P = .01). There were no significant differences according to pathological spontaneous activity.

**Conclusion:**

ADC_mean_ and ADC_min_ showed strong positive correlations with the duration of the MUAP in myositis patients. Both modalities might similarly reflect muscle fiber loss in myositis patients.

## 1 INTRODUCTION

Autoimmune myositis is a heterogeneous group of disorders of unknown etiology and can be classified into the following entities: polymyositis, dermatomyositis, inclusion body myositis, necrotizing autoimmune myositis, and overlap myositis (Dalakas, 2015). The diagnostic approach is multimodal consisting of anamnestic features, clinical examination, serological parameters, needle electromyographic findings, and muscle biopsy (Dalakas, 2015). The magnetic resonance imaging (MRI) has been evaluated to be the most important imaging modality in these patients due to its excellent soft tissue contrast (Leung, 2017; O'Connell et al., 2002). Thus, it is clinically used to detect atrophy of affected muscles, muscle edema, and/or myofasciitis (Dalakas, 2015; O'Connell et al., 2002).

Recently, diffusion‐weighted imaging (DWI) has been evaluated to be a useful imaging tool in several muscle disorders such as tumors, inflammation, and myopathies (Partovi et al., 2015; Qi, Olsen, Price, Winston, & Park, 2008; Ran et al., 2016; Surov & Behrmann, 2014; Surov et al., 2015). Additionally, it can be quantified with the apparent diffusion coefficient (ADC) reflecting the random water motion in tissue (Surov, Meyer, & Wienke, 2017).

Another important diagnostic modality is needle electromyography (EMG), which is widely used in clinical routine (Dalakas, 2015). A fine needle electrode is inserted into the muscle. Then, a signature electrical signal is generated by the motor units (MU) displaying extracellular potential differences in muscle cells belonging to the same alpha motor neuron. Furthermore, the MU action potential (MUAP) is brought about by voluntary muscle contraction. In certain pathological conditions, unprovoked regular signals generated by individual muscle fibers can be assessed as pathological spontaneous activity (SA) (Apartis, 2013). Clinically, EMG is mostly used to rule out possible differential diagnoses like neurogenic disorders and to assess disease activity in myositis patients (Dalakas, 2015). Typically, MUAPs become small in amplitude, short in duration, and polyphasic. Furthermore, muscle fiber degeneration is accompanied by pathological spontaneous activity.

It is yet unclear whether electrodiagnostic information about extracellular potential differences provided by EMG is associated with the random water motion in muscles measured by DWI. Presumably, both modalities might be able to assess disease activity in a similar fashion.

Therefore, the purpose of this study was to elucidate possible associations between DWI and EMG in myositis patients.

## 2 MATERIALS AND METHODS

This retrospective study was approved by the institutional ethic committee and informed consent was waived.

### 2.1 Patients

One hundred and six patients with different muscle disorders were investigated by MRI in our department. Patients were included in this study if they fulfilled the following inclusion criteria:

- if they had a muscle disorder (myositis) confirmed by histopathology;
- MRI was performed with DWI;
- Diseased muscles did not show artifacts on DWI, as signal voids, movement artifacts, and susceptibility artifacts
- EMG was performed within 3 days to the MRI and the video EMG recordings were available

Cases that did not meet the inclusion criteria were excluded from the study. Also, patients with traumatic muscle injury, steroid‐induced myopathy, muscle abscesses, and muscles struck by ischemia or venous thrombosis were excluded.

Altogether, seven patients (four women, 57.14%) were included in this study (Table 1).

**Table 1:** Overview about the patient collective included in this study

Diagnosis	*n*	%
Polymyositis	3	42.86
Overlap myositis	3	42.86
Inclusion body myositis	1	14.29
All	7	100

### 2.2 MRI

In all cases, MRI of the thigh and lower leg was performed using a 1.5‐T scanner (Magnetom Vision Sonata Upgrade, Siemens, Germany). MRI sequences included turbo spin‐echo (TSE) images, T2‐weighted (T2W) fat‐suppressed short tau inversion recovery (STIR) images, half‐Fourier acquisition single‐shot turbo spin‐echo (HASTE) images, T1‐weighted (T1W) spin‐echo (SE) images prior and after intravenous administration of contrast medium.

Diffusion‐weighted images were obtained with a multishot SE‐EPI (echo planar imaging) sequence with b‐values, 0, and 1000 s/mm². Motion‐probing gradient pulses were placed in the three orthogonal planes, and isotropic DW imaging was generated by three orthogonal axes. Sequence parameters were as follows: TR/TE: 5800/68 ms; flip angle: 90°, thickness: 5 mm; matrix size: 128; bandwidth: 2.3 kHz; Imaging Frequency: 63.685; number of averages: 2.

### 2.3 ADC measurement

ADC maps were automatically generated by the implemented software. In all cases, polygonal regions of interest (ROI) were manually drawn on the ADC maps along the contours of the affected muscles on each slice (whole muscle measure). A minimum ADC value (ADC_min_), a mean ADC value (ADC_mean_), and a maximum ADC value (ADC_max_) were estimated of every muscle.

### 2.4 Electromyography

The EMG was recorded by M.K. using a concentric needle electrode (37 mm, 26G, CareFusion, USA) and by a Multiliner Vision (Viasys, Höchberg, Germany) in the Electrophysiology Unit of the Department of Neurology. The number of the analyzed motor unit action potential (MUAP) and its characteristic features (amplitude, duration) were measured in each affected muscle. Pathological spontaneous activity (PSA) was evaluated. Video EMG recordings were analyzed for PSA and MUAP variables in each muscle examined. The investigated muscles were as followed: *n *= 2 (25%) M. tibialis anterior, *n *= 2 M. vastus medialis (25%), and *n *= 4 (50%) M. vastus lateralis, respectively.

### 2.5 Statistical analysis

Statistical analysis and graphics creation were performed using GraphPad Prism (GraphPad Software, La Jolla, CA, USA). Collected data were evaluated by means of descriptive statistics (absolute and relative frequencies). Spearman's correlation coefficient (p) was used to analyze associations between investigated parameters. ADC and clinical subgroups were analyzed by Mann–Whitney test. In all instances, *p* values <.05 were taken to indicate statistical significance.

## 3 RESULTS

**Figure 1:**
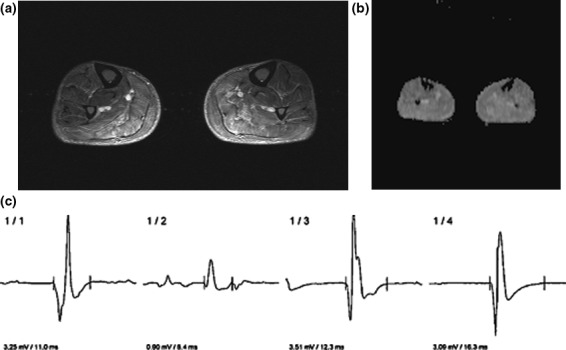
Imaging findings in a 75‐year‐old patient with polymyositis. (a) T2w fat‐suppressed short tau inversion recovery (STIR) image showing edema of the thigh musculature, especially of the triceps surae muscle. The investigated muscle with EMG and ADC is the tibialis anterior muscle. (b) ADC map. The different ADC values of the most affected muscle are as follows: ADC_min_ = 1.32 × 10^−3 ^mm^2^/s, ADC_mean_ = 1.45 × 10^−3 ^mm^2^/s, and ADC_max_ = 1.64 × 10^−3 ^mm^2^/s). (c) The representative EMG curves of the tibialis anterior muscle. The mean MUAP duration is 12.2 ms, and the mean amplitude is 2.69 mV

**Figure 2:**
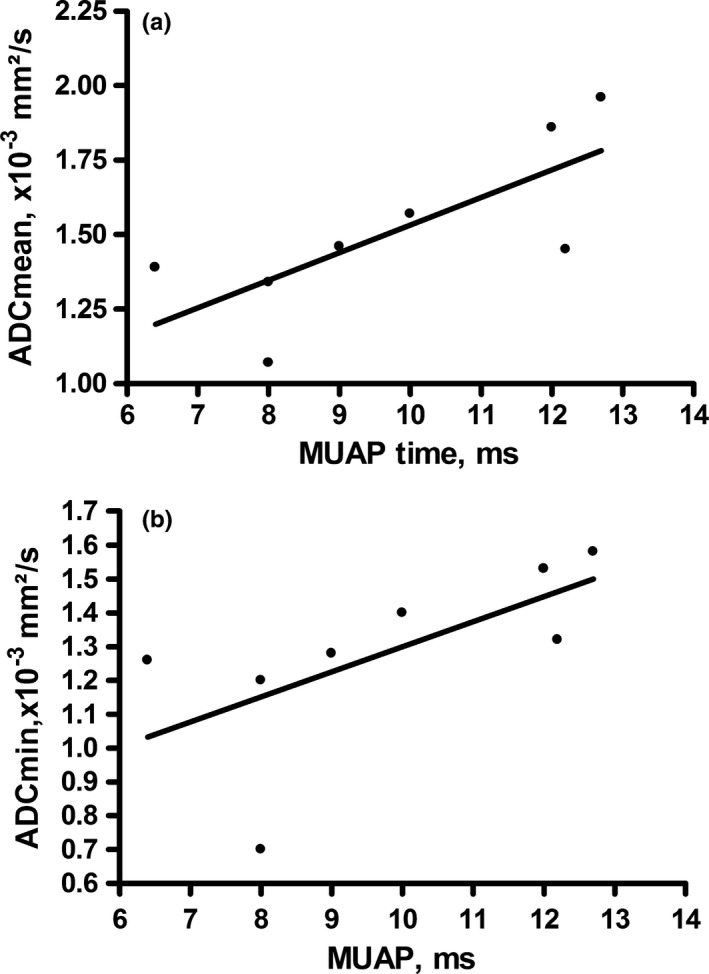
Spearman Correlation analysis identified a positive correlation between ADC_mean_ and the duration of the MUAP, p = .78 P = .0279 and between ADC_min_ and the duration of the MUAP p = .85 P = .01

The estimated mean ADC_mean_ values of the eight muscles were 1.51 ± 0.29 × 10^−3 ^mm²/s, median 1.46 × 10^−3 ^mm²/s, range 1.07–1.96 × 10^−3 ^mm²/s, mean ADC_min_ 1.28 ± 0.27 × 10^−3 ^mm²/s, median 1.30 × 10^−3 ^mm²/s and range 0.70–1.58 × 10^−3 ^mm²/s, and mean ADC_max_ 1.73 ± 0.28 × 10^−3 ^mm²/s, median 1.68 × 10^−3 ^mm²/s and range 1.40–2.20 × 10^−3 ^mm²/s, respectively. Regarding EMG findings, the mean amplitude was 0.97 ± 0.81 mV, and the mean duration was 9.8 ± 2.3 ms. As an illustration, Figure 1 displays a patient of this study collective. Table 2 summarizes the ADC values and EMG findings. Correlation analysis identified significant associations between ADC_mean_ and the duration of the MUAP (p = .78 P = .028) and between ADC_min_ and the duration of the MUAP (p = .85 P = .01) (Table 3, Figure 2).

**Table 2:** Estimated DWI and EMG parameters

Parameters	*M* ± *SD*	Median	Range
ADC_mean_, ×10^−3 ^mm^2^/s	1.51 ± 0.29	1.46	1.07–1.96
ADC_min_, ×10^−3 ^mm^2^/s	1.28 ± 0.27	1.30	0.70–1.58
ADC_max_, ×10^−3 ^mm^2^/s	1.73 ± 0.28	1.68	1.40–2.20
Amplitude, mV	0.97 ± 0.81	0.70	0.31–2.70
Duration, ms	9.79 ± 2.32	9.50	6.40–12.70

**Table 3:** Correlations between DWI and EMG parameters in all patients

Parameters	Amplitude, mV	Duration, ms
ADC_mean_, ×10^−3 ^mm^2^/s	p = .14 P = .75	**p = .78** **P = .0279**
ADC_min_, ×10^−3 ^mm^2^/s	p = .21 P = .62	**p = .85** **P = .01**
ADC_max_, ×10^−3 ^mm^2^/s	p = .02 P = .98	p = .63 P = .10

The statistically significant correlations are highlighted in bold.

Furthermore, we divided the muscles into groups according to pathological spontaneous activity. There were five muscles with pathological spontaneous activity and three without. The ADC values did not differ between these groups (for ADC_mean_ p = .99, for ADC_min_ p = .98, for ADC_max_ p = .79, respectively) (Table 4).

**Table 4:** Muscles divided into groups with pathological spontaneous activity and physiological behavior. There were no significant differences between these groups

ADC parameter	Pathological spontaneous activity (*M* ± *SD*)	No spontaneous activity (*M* ± *SD*)	*p*‐value
ADC_mean_, ×10^−3 ^mm^2^/s	1.55 ± 0.27	1.47 ± 0.29	.99
ADC_min_, ×10^−3 ^mm^2^/s	1.35 ± 0.17	1.24 ± 0.33	.98
ADC_max_, ×10^−3^ mm^2^/s	1.74 ± 0.30	1.72 ± 0.29	.79

## 4 DISCUSSION

To the best of our knowledge, this is the first study investigating possible associations between DWI and EMG findings in myositis patients.

DWI is an imaging modality sensitive to tissue alterations in several muscle disorders (Surov et al., 2015). For example, it has been shown that DWI correlates with cellularity in several tumors (Surov et al., 2017). Moreover, ADC values are significantly different between several muscle tumors reflecting various cellularity in muscle tumors (Surov & Behrmann, 2014; Surov et al., 2015, 2017).

In myositis disorders, the underlying tissue alterations are complex (Dalakas, 2011, 2015). Firstly, T cells invade the endomysium (Dalakas, 2011). Secondly, due to the inflammation reaction muscle fibers degrade by apoptosis and necrosis (Dalakas, 2015). Then, extracellular edema is detectable by morphological MRI (O'Connell et al., 2002). Presumably, the diffusion might be initially restricted due to increasing cellularity by the inflammatory cells. Later, the diffusion might be elevated due to necrosis of the muscle fibers with more free diffusion space for water molecules. Previously, only few studies investigated possible diagnostic value for DWI in myositis (Meyer et al., 2017; Qi et al., 2008; Ran et al., 2016). One study showed a lower ADC value for diseased muscles compared to nondiseased control muscles (Ran et al., 2016), whereas two studies identified a higher ADC value in affected muscles (Meyer et al., 2017; Qi et al., 2008).

Furthermore, the present study is the first to use different ADC fractions. For example, it is commonly mentioned in oncologic imaging that ADC_min_ reflects different microstructural aspects in tumors better than ADC_mean_ or ADC_max_ (Surov et al., 2017). However, in the above‐mentioned myositis studies, only a mean ADC value was used (Meyer et al., 2017; Qi et al., 2008; Ran et al., 2016). In the present study, ADC_max_ did not show significant correlations, whereas ADC_mean_ and ADC_min_ did so. This finding indicates that different ADC fractions may reflect diverse tissue aspects in myositis.

Another important aspect is that the ADC value might change during anti‐inflammatory treatment and could be used as an imaging biomarker, as it is used in several oncologic disorders (Chen et al., 2016; Kyriazi et al., 2011). As a first example, Qi et al. (2008) reported a myositis patient with a decreasing ADC value under treatment. However, further prospective studies are needed to evaluate the ADC value as a possible treatment biomarker.

Regarding EMG, it is clinically widely used in the diagnostic work up in myositis patients (Paganoni & Amato, 2013). Especially, the affected muscles are investigated. Furthermore, it has been shown that EMG can quantitatively reflect muscle alteration (Amato & Barohn, 2009). However, the EMG signal might only display a small area of the muscle and not the muscle as a whole (Stålberg & Karlsson, 2001).

Typical EMG findings in muscles of myositis patients are fibrillations, positive sharp waves, and myotonic discharges (Amato & Barohn, 2009; Paganoni & Amato, 2013). In acute inflammation, a loss of myofibrils leads to MUAPs small in amplitude, short in duration, as well as polyphasic (Amato & Barohn, 2009; Paganoni & Amato, 2013). However, the exact underlying tissue alterations causing these phenomena are still unclear (Stålberg & Karlsson, 2001).

We identified a strong positive correlation between the MUAP duration and ADC_min_ as well as ADC_mean_. MUAP amplitude values did not correlate with the ADC values, although this finding might be at first misleading. Presumably, the loss of muscle fibers leads to higher ADC values due to free diffusion space and a small MUAP. However, the positive correlation might indicate that a lower ADC value reflects the same disease state as a small MUAP. Furthermore, a recent study indicated that the decrease in the duration is more sensitive than the decrease in the amplitude in polymyositis patients (Yang et al., 2014). Based upon the presented data, we hypothesize that a smaller ADC value might indicate a more severe course of myositis than a higher ADC value. As mentioned above, Ran et al. (2016) reported a significantly lower ADC value than the control group, which supports our result.

It is well‐known that EMG correlates with the clinical presentation in myositis patients (Stålberg & Karlsson, 2001; Tymms, Beller, Webb, Schrieber, & Buchanan, 1990). Regarding MRI, a significant correlation was found between clinical presentation and morphological MRI as well as for DWI (Barsotti et al., 2016). Contrarily, another recent study showed no linear correlations between DWI and serological parameters in myositis patients (Meyer et al., 2017).

Regarding electrodiagnostic studies, only one other study investigated possible correlations between ADC and electroneurography in diabetic neuropathy patients (Wu et al., 2017). The authors report a negative correlation coefficient *r* = −.59 between ADC values and motor nerve conduction velocity (Wu et al., 2017). They hypothesized that the velocity is lowered due to demyelination and axonal degeneration, and the diffusion is elevated due to decreasing cellularity (Wu et al., 2017). Comparable with our study, they investigated only a small study sample of 12 patients. In another recent study, possible correlations between DWI and EMG on healthy volunteers could be identified (Surov et al., 2017). It was shown that spontaneous muscle activity can be detected on DWI and surface EMG in a similar fashion, and therefore this study is in good agreement with the present study that both modalities are linked to each other (Schwartz et al., 2018). We hereby present the first data regarding possible associations between ADC and EMG. Based upon these findings, functional MRI, especially DWI, might be able to provide similar information as electrodiagnostic modalities. However, prospective studies with larger patient samples are needed to further elucidate the possible value of functional MRI in myositis. Nevertheless, first results presented here are promising.

There are several limitations of this study. Firstly, it has a retrospective design. Secondly, our patient sample is relatively small, caused by the rarity of this disorder. Thirdly, no patients with dermatomyositis or necrotizing autoimmune myositis could be included in our study sample, which could show different associations. Finally, we acquired DWI with only two b‐values and, therefore, could not calculate other DWI parameters.

In conclusion, there was a strong correlation between ADC_min_ as well as ADC_mean_ and the duration of the muscle unit action potential. DWI and EMG might similarly reflect loss of muscle fibers in myositis patients in a comparable fashion with each other.

## CONFLICT OF INTEREST

The authors declare that they have no conflict of interest. No funding was needed for this research.
